# Generalizable attention U-Net for segmentation of fibroglandular tissue and background parenchymal enhancement in breast DCE-MRI

**DOI:** 10.1186/s13244-023-01531-5

**Published:** 2023-11-06

**Authors:** Sylwia Nowakowska, Karol Borkowski, Carlotta M. Ruppert, Anna Landsmann, Magda Marcon, Nicole Berger, Andreas Boss, Alexander Ciritsis, Cristina Rossi

**Affiliations:** 1 Diagnostic and interventional Radiology, University Hospital Zurich, University Zurich, Rämistrasse 100, 8091 Zurich, Switzerland; 2 b-rayZ AG, Wagistrasse 21, 8952 Schlieren, Switzerland; 3 Present Address: Institut RadiologieSpital Lachen, Oberdorfstrasse 41, 8853 Lachen, Switzerland; 4 Present address: GZO AG Spital Wetzikon, Spitalstrasse 66, 8620 Wetzikon, Switzerland

**Keywords:** Fibroglandular tissue segmentation, Background parenchymal enhancement segmentation, Deep learning, Breast MRI, Assessment standardization

## Abstract

**Objectives:**

Development of automated segmentation models enabling standardized volumetric quantification of fibroglandular tissue (FGT) from native volumes and background parenchymal enhancement (BPE) from subtraction volumes of dynamic contrast-enhanced breast MRI. Subsequent assessment of the developed models in the context of FGT and BPE Breast Imaging Reporting and Data System (BI-RADS)-compliant classification.

**Methods:**

For the training and validation of attention U-Net models, data coming from a single 3.0-T scanner was used. For testing, additional data from 1.5-T scanner and data acquired in a different institution with a 3.0-T scanner was utilized. The developed models were used to quantify the amount of FGT and BPE in 80 DCE-MRI examinations, and a correlation between these volumetric measures and the classes assigned by radiologists was performed.

**Results:**

To assess the model performance using application-relevant metrics, the correlation between the volumes of breast, FGT, and BPE calculated from ground truth masks and predicted masks was checked. Pearson correlation coefficients ranging from 0.963 ± 0.004 to 0.999 ± 0.001 were achieved. The Spearman correlation coefficient for the quantitative and qualitative assessment, i.e., classification by radiologist, of FGT amounted to 0.70 (*p* < 0.0001), whereas BPE amounted to 0.37 (*p* = 0.0006).

**Conclusions:**

Generalizable algorithms for FGT and BPE segmentation were developed and tested. Our results suggest that when assessing FGT, it is sufficient to use volumetric measures alone. However, for the evaluation of BPE, additional models considering voxels’ intensity distribution and morphology are required.

**Critical relevance statement:**

A standardized assessment of FGT density can rely on volumetric measures, whereas in the case of BPE, the volumetric measures constitute, along with voxels’ intensity distribution and morphology, an important factor.

**Key points:**

• Our work contributes to the standardization of FGT and BPE assessment.

• Attention U-Net can reliably segment intricately shaped FGT and BPE structures.

• The developed models were robust to domain shift.

**Graphical Abstract:**

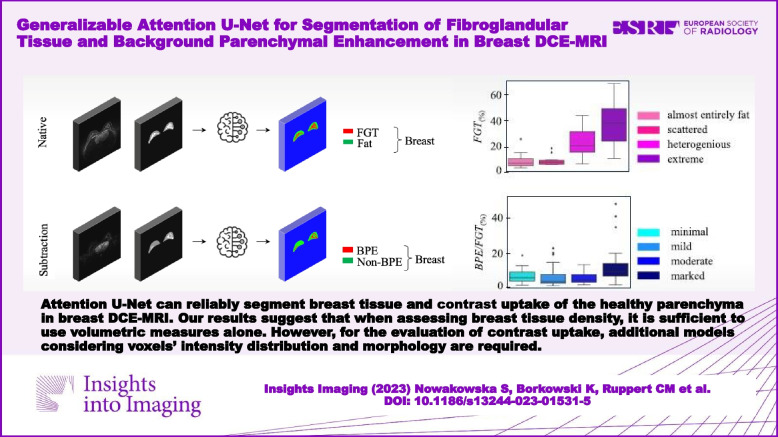

**Supplementary Information:**

The online version contains supplementary material available at 10.1186/s13244-023-01531-5.

## Introduction

Breast cancer is the most common cancer in the female population worldwide [1]. Screening and early detection followed by treatment are indispensable to improving survival rates. Due to the limitations of mammography, MRI examinations are recommended for high-risk patients and for patients with extremely dense breasts [2]. The amount of fibroglandular tissue (FGT) and the contrast uptake of the healthy FGT, i.e., background parenchymal enhancement (BPE), are important factors related to breast cancer risk, diagnosis, and management [3–7]. For example, the study performed by Ray et al. demonstrated that high BPE was linked to higher rates of abnormal interpretation and biopsy procedures as well as lower specificity [7]. The findings of the study by Hu et al. indicate that elevated BPE is correlated with an increased risk of breast cancer [4]. This is why the American College of Radiology (ACR) in the fifth edition of the Breast Imaging Reporting and Data System (BI-RADS) for MRI reporting recommends visual estimation of the FGT density followed by its classification into four categories: (a) *almost entirely fat*, (b) *scattered*, (c) *heterogeneous*, and (d) *extreme*. The BPE, after visual assessment, should be classified into (a) *minimal*, (b) *mild*, (c) *moderate*, and (d) *marked* categories [8]. As shown by different studies, such a qualitative assessment is prone to inter-reader variability. The reported Cohen’s kappa values for BPE assessment range from 0.3 (fair agreement) to 0.95 (almost perfect agreement) [9]. This is the reason why currently there are ongoing efforts to standardize the FGT and BPE assessment by quantitative measures. The most commonly used approaches are based on a region of interest selected by the user, which is prone to inter-reader variability, and on segmentation of the whole structures followed by their quantification [10]. Currently, the ACR is awaiting more robust data for the recommendation of quantitative assessments in clinical practice [8]. To achieve this goal, highly reliable and robust algorithms segmenting FGT and BPE are indispensable.

The main challenge is that breast MRI acquisition is not standardized: different institutions and doctors use different equipment, magnetic field strength, protocols (including the timing and the amount of the post-contrast acquisitions), different contrast agents, and patient positioning, to acquire and process the data. As a result, MRI data exhibit different resolution, contrast, noise level, and artifacts leading to high variability in the quality and reliability of the diagnosis.

Over the past few years, the field of CNN-based segmentation models for medical imaging data has witnessed remarkable advancements. Among these, the U-Net architecture [11] and its various adaptations [12–18] have been developed. A particularly noteworthy enhancement to the U-Net family is the attention U-Net [19], which integrates attention gates into the architecture. This enables the model to efficiently concentrate on essential features while ignoring irrelevant regions.

It has been shown that the CNN-based segmentation architectures can reliably segment the FGT tissue from native MRI sequences, with the DSC values ranging from 0.81 ± 0.11 to 0.87 ± 0.08 [20–25]. Regarding the BPE segmentation, the reported approaches rely on the FGT segmentation from native images with subsequent transfer of the resulting mask to subtraction images, followed by the BPE segmentation based on mean and standard deviation of the voxels’ intensity values [22, 26] or on a predefined threshold [27, 28]. It should be noted that voxels’ intensity values in the MRI data are dependent on the magnetic field strength and the scanner hardware as well as on many adapted parameters during data acquisition and post-processing [29]. Moreover, the voxels’ intensity values in the subtraction data depend on the amount of the contrast agent and the timing of the acquisition. Additionally, the slices acquired at the breast periphery feature higher intensity than slices acquired in the middle of the breast. Thus, BPE segmentation directly from subtraction volumes is desired. In our previous work [30], we showed promising results using U-Net architecture and data from a single scanner for training, validation, and testing. However, segmentation of FGT and testing on an external dataset was not included in this study.

The primary objective of the first part of this study was to develop two automated and generalizable segmentation models: one segmenting the FGT from native volumes, the other segmenting the BPE from subtraction volumes. In the second part of the study, the resulting models were applied to 80 DCE-MRI examinations of 75 patients from our institution, with the aim to assess the correlation between the volumes of FGT and BPE and classes assigned visually by radiologists.

## Materials and methods

### Patient data

This retrospective study has been approved by the local ethics committee. The main parameters of the datasets utilized in this study are provided in Table 1. Datasets 1–3 originate from our institution, whereas dataset 4 is a small subset of public Duke-Breast-Cancer Dataset [31]. All the data was acquired in a transverse plane in a prone position with fat-saturation of the DCE T1 sequences.

**Table 1:** Main parameters of the three datasets used in this work

	**Dataset 1**	**Dataset 2**	**Dataset 3**	**Dataset 4**
Institution	Our institution	Our institution	Our institution	Duke dataset
Acquisition time range	September 2013–October 2015	September 2020–October 2022	June 2018–October 2022	January 2000–March 2014
Magnetic field strength [T]	3.0	1.5	3.0	3.0
Manufacturer and model	Siemens, Magnetom Skyra	Siemens, Sola	Siemens, Magnetom Skyra	Siemens, Magnetom Skyra
Resolution [px × px]	448 × 448	384 × 384	448 × 448	448 × 448
TR [ms]	4.40–4.64	4.98	4.40–4.48	3.77
TE [ms]	1.56–1.78	2.39	1.62–1.70	1.44
Flip angle [°]	10	10	10	10
FGT model: patients (cases)/volumes/slices	29/29/3048	4/4/436	71/76/9088	3/3/672
BPE model: patients (cases)/volumes/slices	82/169/17420	4/4/1744	71/76/9088	3/6/1344
Patients’ age mean ± std [years]	47.3 ± 11.6	40.3 ± 19.4	48.8 ± 12.6	52.6 ± 6.6
Use in the study	Patient stratified train/valid/test split	Test set Volumetric analysis	Volumetric analysis	Test set

To curate datasets 1–3, we conducted a search in the Picture Archiving and Communication System (PACS) of our institution. The search aimed to identify DCE-MRI examinations fulfilling following inclusion criteria: (a) age above 18 years, (b) absence of implants, (c) availability of assessments for FGT and BPE classes in the corresponding radiological report, and (d) BI-RADS Assessment Category indicating a likelihood of malignancy of 1 or 2, corresponding to a 0% likelihood [8]. Examinations lacking proper fat saturation or suffering from motion blur were excluded. The FGT and BPE classes, determined via a board-certified radiologist, were extracted from the radiological report.

Dataset 1 was curated by searching through examinations acquired between September 2013 and October 2015 using a 3.0-T scanner. This dataset was used for model training, validation, and testing. For volumetric analysis, the search through examinations acquired between September 2020 and October 2022 continued until a total of 80 eligible examinations resulting in possibly well-equalized distributions of FGT and BPE classes were identified. In this way, dataset 2, containing examinations acquired with 1.5-T scanner, and dataset 3, containing examinations acquired with 3.0-T scanner, were obtained. Examinations from dataset 2 were additionally used for testing models’ performance.

### AI model development design

The design of AI model development differed from previous works by utilizing two separate models: one for FGT segmentation from the native volume, the other one for BPE segmentation from the subtraction volumes. This approach allows for an investigation of all subtraction images, without the need for precise registration. This is important, as even a small misalignment between sequences (cf. Fig. S1) can have a significant impact on volumetric analysis, especially in cases with *almost entirely fat* FGT and *minimal* BPE. Another advantage of using two separate models is that potential errors in FGT segmentation do not affect BPE segmentation. For instance, artifacts or extreme superior and inferior regions with higher intensity on native volume may be mistakenly included in the FGT segmentation.

**Fig. 1:**
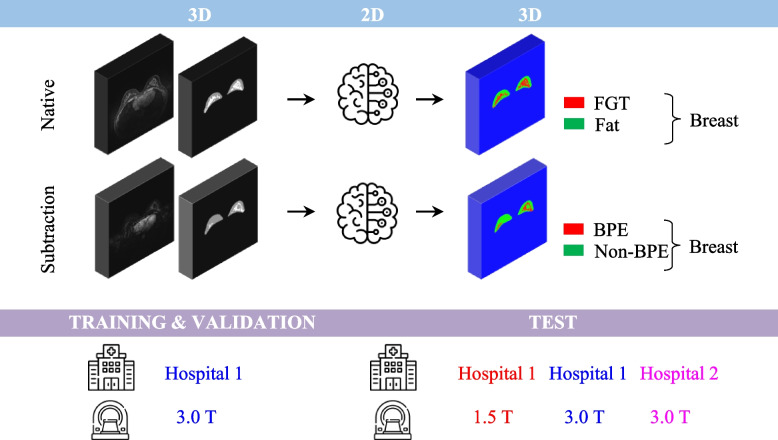
Schematic representation of the model development pipeline. Two independent attention U-Net models are trained: the first one is trained to segment the fibroglandular tissue (FGT) and the fatty tissue from native DCE data; the second one is trained to segment BPE and non-enhancing tissue from the subtraction data. This separation ensures accurate segmentation even for not well-registered cases. In both cases, the segmentation is performed slice-wise ensuring that with the chosen hardware, the predicted mask has high resolution able to accurately capture the intricate details of the FGT and BPE structures (Icons made by Freepik and Netscript from flaticon.com)

As the FGT and BPE are very complex and fine structures, it is important that the predicted mask has similar resolution to the resolution with which the MRI data was acquired. Hence, we opted for the highest resolution of our data, i.e., 448 × 448, as the input and output size. To accommodate a reasonable batch size for training with NVIDIA GeForce RTX 3090 (24 GB), we chose to train the models slice by slice (Fig. 1). Our approach utilized a 2D implementation of the attention U-Net from the repository of Yingkai Sha [32] with spatial 2D dropout layers added in each convolution stack. The additional advantage of using a 2D model is that it can effectively incorporate volumes with varying numbers of slices and any necessary rescaling is performed solely in 2D.

### Ground truth masks

The ground truth masks were created in 3D Slicer [33] by S.N.. Firstly, the breast was segmented without skin by the use of Grow from Seeds algorithm, Gaussian smoothing, and fine-tuning with Paint and Erase. Afterwards, the FGT and BPE were segmented by thresholding, customized to each volume followed by the fine-tuning allowing also for artifacts’ removal. A sample of final segmentation masks was verified by A.L. (resident in radiology with more than 3 years of experience in breast imaging) and A.B. (board-certified radiologist with over 15 years of experience in breast imaging). Due to the time and resource constrains, intra- and inter-reader variability investigation was not performed.

### Dataset splitting

Dataset 1 was split into a patient-stratified train-validation-test sets, and the FGT model was trained using 2112 slices from 20 patients for training, 416 slices from 4 patients for validation, and 520 slices from 5 patients for testing. The model was additionally evaluated on dataset 2 and dataset 4, which comprised 1004 slices from 6 patients. Importantly, the subtraction volumes exhibit lower contrast and signal-to-noise ratio compared to native volumes. To account for this, a larger amount of data was utilized for the BPE model. The total training set for the BPE model comprised 11,829 slices from 54 patients, with a validation set of 2469 slices from 12 patients, and a testing set of 3095 slices from 16 patients. Subsequently, the BPE segmentation model was tested on dataset 2 and dataset 4, which collectively contained 2672 slices from 6 patients.

### Model training

All the data were rescaled to 0–1 range prior to training. A subset of the dataset was used for hyperparameter tuning using fivefold cross validation. The best hyperparameters obtained in this way were then fine-tuned during training using the entire dataset. Five rounds of training of native and subtraction models were then performed using best fine-tuned hyperparameters. Noteworthy, the best performance was achieved with focal Tversky loss [34] function harshly penalizing the false negatives by setting the *α* parameter of the loss to 0.99 and the *β* parameter to 0.01. Additionally, during training, brightness augmentation in the 0.2–1.8 range delivered best performance on the test set. All final hyperparameters together with average inference runtimes are reported in Table S1.

### Model evaluation

The model obtained in each training run was evaluated on the test data coming from three datasets: dataset 1, 2, and 4 (cf. Table 1). Firstly, the evaluation was centered around application-relevant metrics, i.e., *breast*_*vol*_, *FGT*_*(%)*_ (1)/*BPE*_*(%)*_ (2), derived from ground truth and predicted masks.

1 $${FGT}_{(\%)}=\frac{{FGT}_{vol}}{{breast}_{vol}} 100\%$$

2 $${BPE}_{(\%)}=\frac{{BPE}_{vol}}{{breast}_{vol}} 100\%$$

Their correlation was plotted, followed by linear fit and calculation of Pearson correlation coefficient (*r*). Secondly, the volumetric DSC was computed for the breast and the FGT/BPE masks. Additionally, a weighted DSC was calculated, with the weights proportional to *FGT*_*(%)*_/*BPE*_*(%)*_. This adjustment was made to account for the higher penalization of small shifts in case of lower *FGT*_*(%)*_/*BPE*_*(%)*_. The models were additionally evaluated with Bland–Altman plots. Lastly, the overlays of the ground truth and the predicted masks were assessed visually.

### Volumetric analysis

The best performing models were used to quantify the density of the healthy breast tissue and its percentage taking up the contrast using datasets 2 and 3. *FGT*_*(%)*_ according to Eq. (1) and *BPE/FGT *_*(%)*_ according to Eq. (3) were calculated from the predicted masks.

3 $${BPE/FGT}_{(\%)}=\frac{{BPE}_{vol}}{{FGT}_{vol}} 100\%$$

Next, the correlation between those quantitative measures and qualitative assessment by radiologists was assessed by using Spearman correlation coefficient (*ρ*), taking into account errors in the calculation of *FGT*_*(%)*_ and *BPE/FGT*_*(%)*._ These were calculated by propagation of uncertainty from mean absolute errors of the native and subtraction model using the test set.

All of the evaluation was performed by S.N..

## Results

### Evaluation of the FGT and BPE segmentation models

The performance metrics of the models trained with the best fine-tuned hyperparameters is reported in Table 2. The *r* for the native models amounted to 0.999 ± 0.001 for the breast and 0.985 ± 0.001 for the *FGT*_*(%)*_*.* These models were characterized by DSC of 0.950 ± 0.002 for the breast segmentation and by DSC and weighted DSC of 0.820 ± 0.005 and 0.864 ± 0.004 for the FGT segmentation, respectively. The *r* for the subtraction models amounted to 0.992 ± 0.001 for the breast and 0.963 ± 0.004 for the *BPE*_*(%)*_*.* These models featured DSC of 0.927 ± 0.001 for the breast segmentation and DSC and weighted DSC of 0.628 ± 0.018 and 0.715 ± 0.015 for the BPE segmentation, respectively.

**Table 2:** The evaluation of native and subtraction models; the mean and standard deviation calculated from 5 training rounds is reported for each metrics in each case

		***r***^**a**^	**DSC**	**Weighted DSC**
Native	Breast	0.999 ± 0.001	0.950 ± 0.002	-
	FGT	0.985 ± 0.001	0.820 ± 0.005	0.864 ± 0.004
Subtraction	Breast	0.992 ± 0.001	0.927 ± 0.001	-
	BPE	0.963 ± 0.004	0.628 ± 0.018	0.715 ± 0.015

^a^Pearson correlation coefficient between the volumes of the *breast*_*vol*_, *FGT*_*(%)*_ (cf. Eq. (1)), *BPE*_*(%)*_ (cf. Eq. (2)) derived from ground truth and predicted masks. The *p*-values are all below 0.0001

**Fig. 2:**
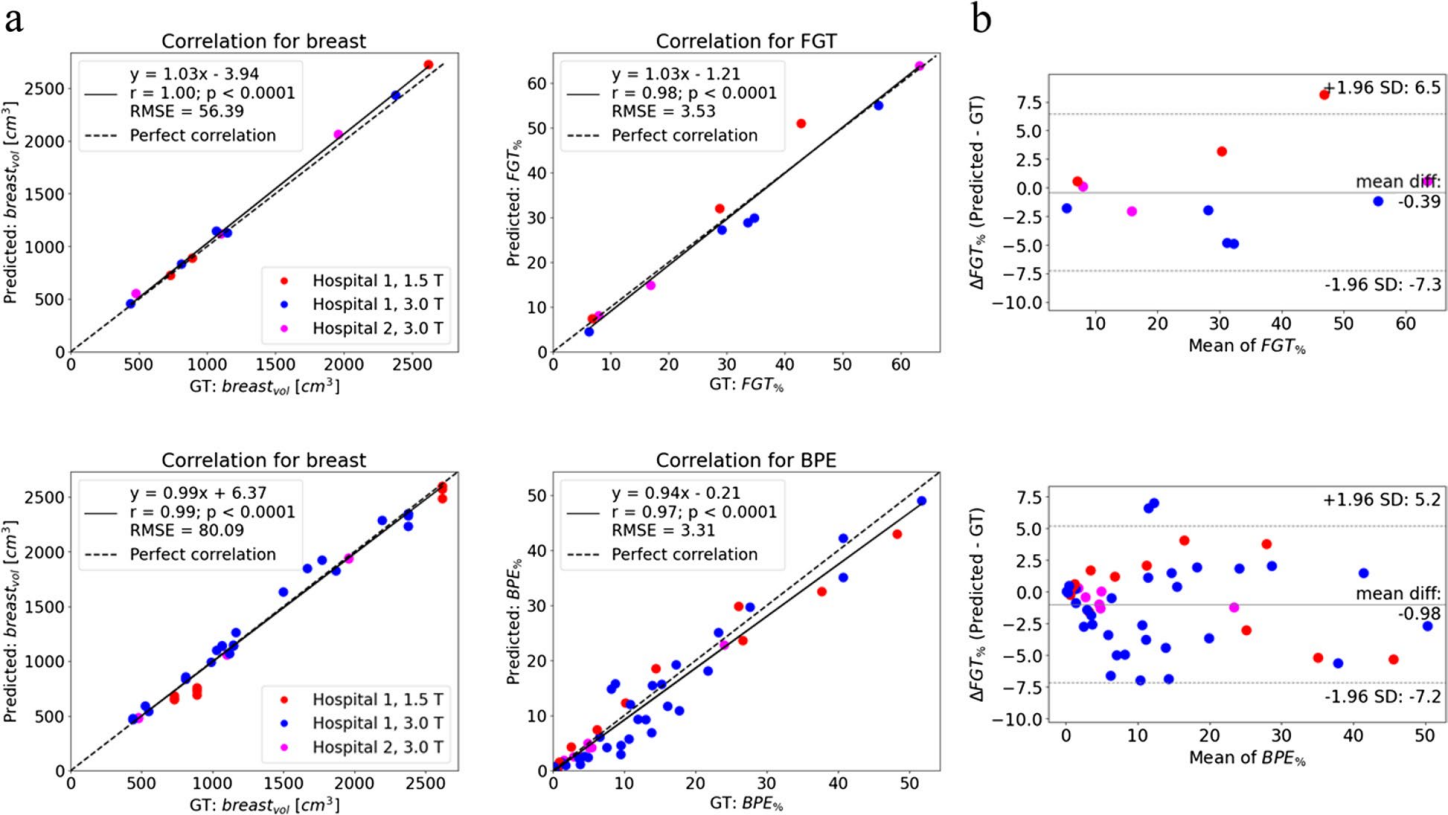
Analysis of the volumetric correlations between ground truth and predicted masks for the test set. **a** 1st row: correlation plots for the chosen native model for *breast*_*vol*_ and *FGT*_*(%)*_ (for 11 volumes). 2nd row: correlation plots for the chosen subtraction model for *breast*_*vol*_ and *BPE*_*(%)*_ (for 49 volumes). **b** Bland-Altmann plots for the chosen native and subtraction models

**Fig. 3:**
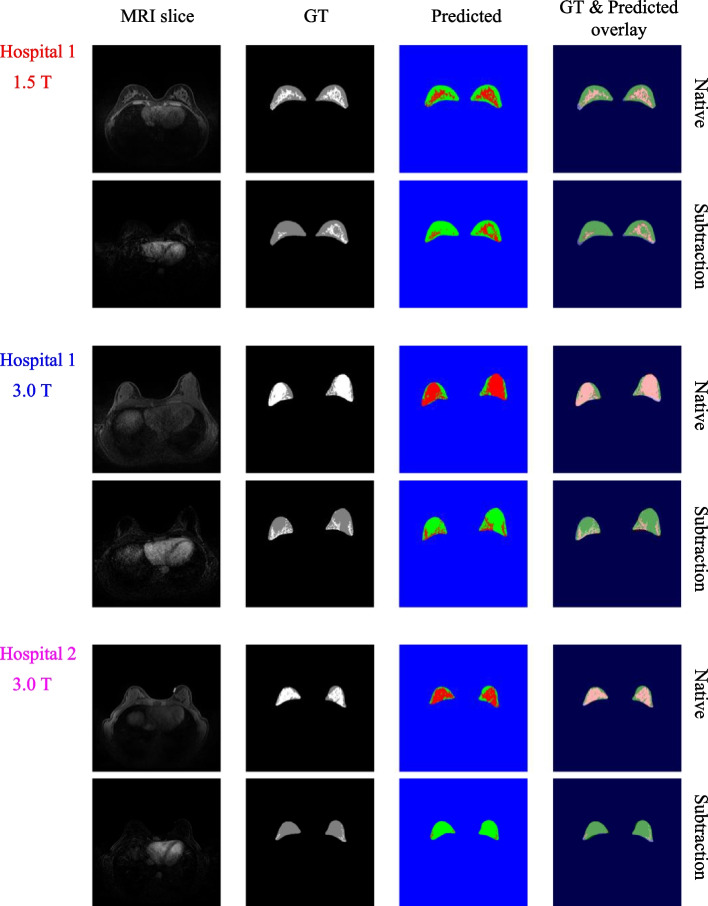
Visual comparison of the overlay between ground truth and predicted masks for a chosen native slice and its corresponding subtraction slice from each dataset

For further analysis, one native model and one subtraction model was chosen. The choice was made based on *r* and DSC values as well as the visual assessment. Analysis of the volumetric correlations between ground truth and predicted masks for the test set for the chosen models is displayed in Fig. 2. The fit regression lines are close to lines describing a perfect correlation. The chosen native model featured DSC of 0.950 (0.937–0.963, 95% confidence intervals (CI)) for the breast, and 0.824 (0.760–0.888, 95% CI) for the FGT. The chosen subtraction model was characterized by DSC of 0.923 (0.924–0.935, 95% CI) for the breast and 0.655 (0.614–0.696, 95% CI) for BPE. The Bland–Altman plots revealed a slight bias of − 0.39% in the estimation of *FGT*_*%*_ with the limits of agreements (LoA) of − 7.3% and 6.5%. The bias in the estimation of *BPE*_*%*_ amounted to − 0.98% with LoA of − 7.2% and 5.2%. Figure 3 showcases the overlays obtained for three sample native and corresponding subtraction slices, each originating from a different dataset. These models were then used for segmenting volumes from dataset 3.

### Results of volumetric analysis

**Fig. 4:**
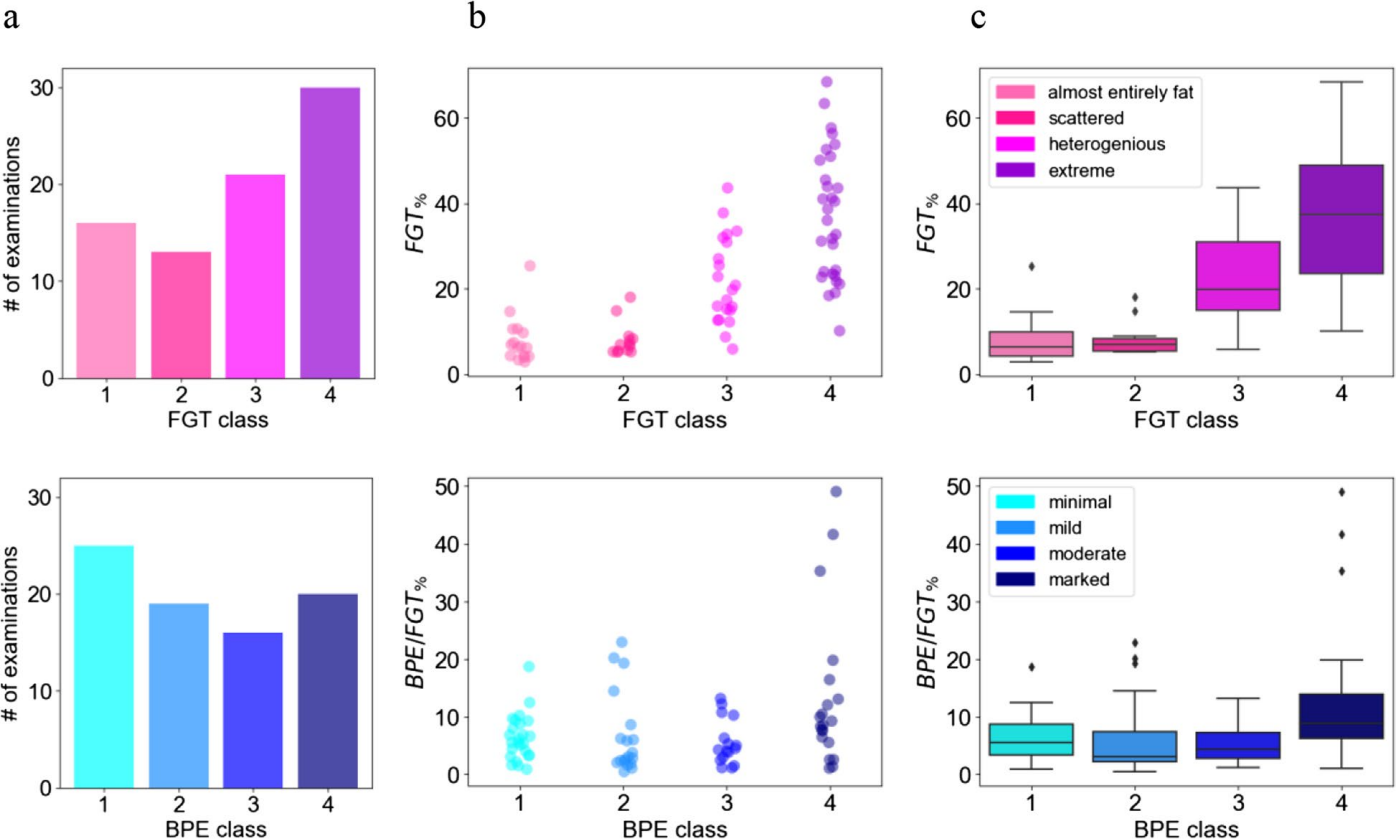
Comparison of the quantitative measures and qualitative assessments by the radiologists. **a** The distributions of the BPE and FGT classes. **b** Plot of calculated *FGT*_*(%)*_ and *BPE/FGT*_*(%)*_ from the predicted masks for each class. **c** The corresponding box plots

Figure 4 depicts the comparison of quantitative measures, i.e., *FGT*_*(%)*_ and *BPE/FGT*_*(%)*_, calculated from the predicted masks with the qualitative classification into four classes by the radiologists. In the case of *FGT*_*(%)*_ distribution, the mean values increase with the increasing FGT class, which is not the case for the mean *BPE/FGT*_*(%)*_ values and the increasing BPE class. The *FGT*_*(%)*_ distribution of *almost entirely fat* and *scattered* FGT classes fully overlap. This is also the case for *BPE/FGT*_*(%)*_ in case of *minimal*, *mild*, and *moderate* BPE classes. The coefficient ρ for *FGT*_*(%)*_ amounted to 0.70 (*p* < 0.0001), whereas for *BPE/FGT*_*(%)*_ amounted to 0.37 (*p* = 0.0006).

## Discussion

The attention U-Net models developed in this study accurately segmented FGT and BPE structures from breast DCE-MRI data. Table 3 presents a summary of the chosen native and BPE models’ performance compared to other studies focused on BPE segmentation using CNN-based models. However, due to the variability in approaches and datasets utilized across studies, a direct comparison is not possible. Owing to the separation of segmentation from native and subtraction volumes, the models developed in this study are applicable to DCE examinations, in which the series are not perfectly registered, opening the possibility to study the volumetric changes also in the further subtraction sequences. Additional advantage is that errors in the FGT segmentation, such as inclusion of high-intensity artifacts, do not affect the BPE segmentation.

**Table 3:** Comparison of our work with other studies concerning with BPE segmentation with CNN-based models

REF	Approach	Model	Dataset	MRI scanners	DSC	*r*	Comment
21	Segmentaion of FGT from native images, transfer of the mask to subtraction images. Segmentation of BPE based on mean and std of intenisty values	3D V-Net	794 patients with unilateral breast cancer (healthy breast was segmented)	3.0 T: Siemens Verio Phillips Ingenia 1.5 T: GE Signa	Breast 0.91 ± 0.04 FGT 0.85 ± 0.11	Breast: 0.96 FGT: 0.93	3.0 T Siemens and Phillips data in the training and testing set, seprate test set with GE 1.5 T data Evaluation of BPE segmentation not reported
29	Segmentation of BPE from subtraction images	2D U-Net	38 patients (slices not depicting tumor)	3.0 T: Siemens Skyra	Overall: 0.76	-	Only BPE segmentation
Our work	Segmentation of FGT from native images, independent segmentation of BPE from subtraction images	2D attention U-Net	88 patients (slices not depicting tumor)	1.5 T: Siemens, Sola 3.0 T: Siemens Skyra (two hospitals)	FGT model: Breast 0.950 ± 0.002 FGT 0.820 ± 0.005 (0.864 ± 0.004 wDSC) BPE model: Breast 0.927 ± 0.001 BPE 0.628 ± 0.018 (0.715 ± 0.015 wDSC)	FGT model: Breast 0.999 ± 0.001 FGT_%_ 0.985 ± 0.001 BPE model: Breast 0.992 ± 0.001 BPE_%_ 0.963 ± 0.004	Data coming from only one scanner used for model training and validation

The comparison of quantitative measures and qualitative assessment of breast tissue density revealed a correlation characterized by *ρ* = 0.70 (*p* < 0.0001), which is statistically strong. The box plots summarizing the *FGT*_*(%)*_ distribution for each class (cf. Figure 4c, upper row) reveal the possibility of FGT class assessment based solely on the volumetric measures. This implies that breast DCE-MRI data from different institutions could be merged, and subsequently distinct and non-overlapping *FGT*_*(%)*_ ranges for each class could be defined, thus allowing reproducible and standardized breast density assessment. This could be further explored in the investigation of breast cancer risk factors enabling triaging of the patients.

It is crucial to note that the statistically low correlation between quantitative and qualitative assessment of contrast uptake (*ρ* = 0.37, *p* = 0.0006, *cf*. Figure 4c lower row) highlights the complexity of its evaluation and underlines the need for an additional model, taking into account the voxels’ intensity values distribution and morphology [10] as well as the sensitivity of radiologists to a potential masking effect for the standardized assessment.

In the context of the four-class BI-RADS-compliant classification of the BPE, few approaches have been explored. Borkowski et al. reported a direct classification of the entire MRI slice by CNN [35]. Nam et al. extracted radiomic features from the BPE, segmented on the basis of mean and standard deviation of voxels’ intensity values in the subtraction image within the FGT mask, followed by the classification by a tree-based model [22]. The classification accuracy in both of these approaches could potentially be improved by implementing the BPE segmentation model developed in this work, thus bringing the models closer to implementation into clinical practice.

We are also aware of the limitations of our study. All the MRI scanners used in our study were from a single manufacturer, and dataset 2 and dataset 4 had a limited number of MRI scans. We are currently working to expand our test dataset to address these limitations.

## Conclusion

The findings presented in this study highlight that a standardized assessment of FGT can rely solely on volumetric measures, while standardized assessment of BPE requires additional models that consider the distribution of intensity and morphology within enhancing voxels.

Due to the impracticality of annotating vast amounts of data from various MRI scanners, it is essential to develop segmentation models trained with limited data yet robust to domain shifts. Our work presents an end-to-end pipeline that creates generalizable models capable of accurately segmenting intricately shaped FGT and BPE structures from native and low-contrast subtraction volumes. Our approach can be extended to other MRI protocols.

### Supplementary Information

**Additional file 1: Figure S1.** The registration issue between native and four contrast-enhanced sequences in DCE-MRI: with the progress of the data acquisition the subtraction slices are progressively shifted. As a result, the FGT mask does not include all the enhancing tissue. This can lead to underestimation of BPE_(%)_, which might be particularly significant in case of entirely fat FGT and minimal BPE. **Table S1.** Overview of the Data, Hardware, Best Hyperparameters and Inference Time for the models reported in the main text.

## Data Availability

The dataset generated and analyzed during the current study is not publicly available due to the confidentiality of the patient data as stated in the Study Plan approved by the Ethics Committee of Kanton of Zürich. Part of the dataset can be made available from the corresponding author to bona fide researchers for non-commercial purposes on reasonable request.

## Abbreviations

ACR: American College of Radiology
BI-RADS: Breast Imaging Reporting and Data System
BPE: Background parenchymal enhancement
CI: Confidence intervals
CNN: Convolutional neural networks
DCE-MRI: Dynamic contrast-enhanced magnetic resonance imaging
DSC: Dice similarity coefficient
FGT: Fibroglandular tissue
LoA: Limits of agreement
PACS: Picture Archiving and Communication System
ρ: Spearman correlation coefficient
r: Pearson correlation coefficient
