# Supplementary material for: Generalizable attention U-Net for segmentation of fibroglandular tissue and background parenchymal enhancement in breast DCE-MRI
Source: Insights Imaging. 2023 Nov 6;14:185. doi: 10.1186/s13244-023-01531-5 (PMC10628070; doi:10.1186/s13244-023-01531-5)
Supplement: Supplementary file 1 — Additional file 1: Figure S1. The registration issue between native and four contrast-enhanced sequences in DCE-MRI: with the progress of the data acquisition the subtraction slices are progressively shifted. As a result, the FGT mask does not include all the enhancing tissue. This can lead to underestimation of BPE(%), which might be particularly significant in case of entirely fat FGT and minimal BPE. Table S1. Overview of the Data, Hardware, Best Hyperparameters and Inference Time for the models reported in the main text. [file 13244_2023_1531_MOESM1_ESM.docx]

**Generalizable Attention U-Net for Segmentation of Fibroglandular Tissue and Background Parenchymal Enhancement in Breast DCE-MRI**

**ELECTRONIC SUPPLEMENTARY MATERIAL**


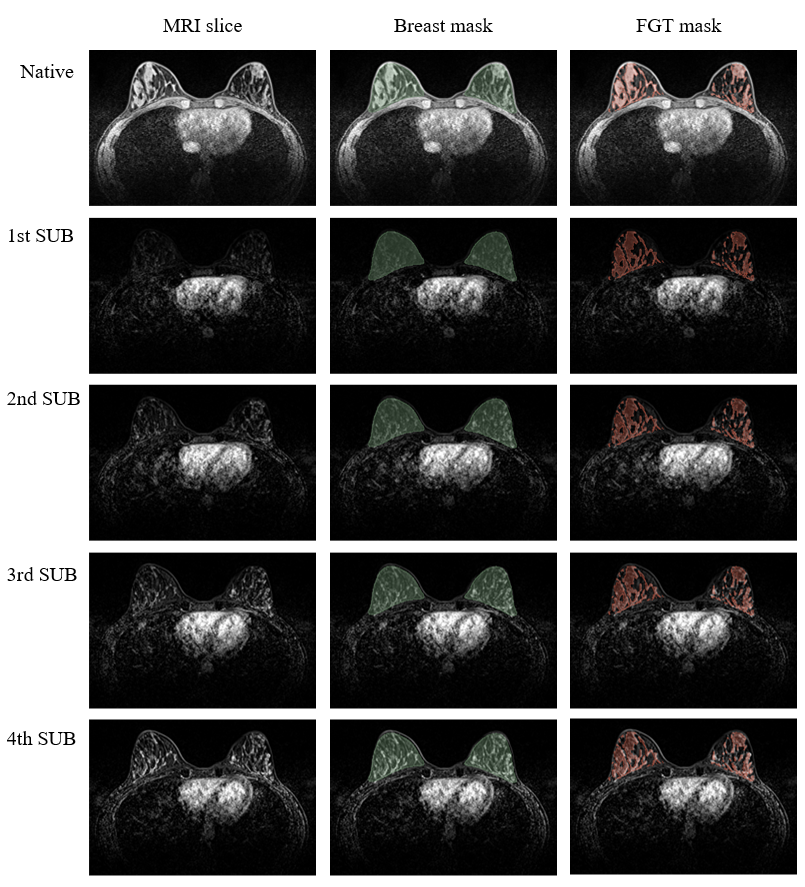


**Figure S1.** The registration issue between native and four contrast-enhanced sequences in DCE-MRI: with the progress of the data acquisition the subtraction slices are progressively shifted. As a result, the FGT mask does not include all the enhancing tissue. This can lead to underestimation of BPE_%_, which might be particularly significant in case of *entirely fat* FGT and *minimal* BPE.

**Table S1.** Overview of the Data, Hardware, Best Hyperparameters and Inference Time for the models reported in the main text.

|  | **FGT model** | **BPE model** |
| --- | --- | --- |
| Patients / Slices | 35 / 4052 | 88 / 20092 |
| GPU | NVIDIA GeForce RTX 3090  24 GB | NVIDIA GeForce RTX 3090  24 GB |
| Runtime [h] | 4 | 16 |
| # of filters for down- & up-sampling blocks | [62, 128, 256, 512, 1024] | [62, 128, 256, 512, 1024] |
| # of conv layers per down- & up-sampling block | 2 | 2 |
| Attention operation | Multiply | Multiply |
| Spacial2D Dropout | 0.20 | 0.20 |
| Learning rate | 1E-5 | 1E-5 |
| Focal Tversky Loss: α, β | FocalTversky: 0.99, 0.01 | Focal Tversky: 0.99, 0.01 |
| Augmentation Brightness range | 0.2 – 1.8 | 0.2 – 1.8 |
| Inference time [s]  volume with 104 slices | 6.25 ± 0.05 | 6.26 ± 0.05 |
| Inference time [s]  volume with 256 slices | 15.72 ± 0.49 | 15.40 ± 0.24 |

**Section S1. Model Training, Inference, and Evaluation**

The overview data, hardware, hyperparameters tunning, and inference time for the models reported in the main text is presented in Table S1.

All the models were trained with Batch Normalization, MaxPooling2D in downsampling blocks, and Upsampling2D with bilinear interpolation in upsampling blocks. ReLu was used as an Activation and Attention Activation function.

Adam optimizer was used. The maximum number of epochs was set to 100 – 150 with patience set to 12.

To ensure stable training the maximum batch size of 10 slices was used.

During each training following augmentation operations were used: zooming, horizontal and vertical shifts in
0.0 – 0.1 range as well as horizontal flipping.
